# Socioeconomic Changes and Adolescent Psychopathology in a Brazilian Birth Cohort Study

**DOI:** 10.1016/j.jadohealth.2012.06.026

**Published:** 2012-12

**Authors:** Luciana Anselmi, Ana M.B. Menezes, Pedro C. Hallal, Fernando Wehrmeister, Helen Gonçalves, Fernando C. Barros, Joseph Murray, Luis A. Rohde

**Affiliations:** a Postgraduate Program in Epidemiology, Federal University of Pelotas, Pelotas, Brazil; b Postgraduate Program in Health and Behavior, Catholic University of Pelotas, Pelotas, Brazil; c Department of Psychiatry, University of Cambridge, Cambridge, United Kingdom; d Child and Adolescent Psychiatric Division, Department of Psychiatry, Federal University of Rio Grande do Sul and National Institute of Developmental Psychiatry for Children and Adolescents, Federal University of São Paulo, Brazil

**Keywords:** Psychopathology, Income, Poverty, Socioeconomic status, Conduct disorders, Emotional disorders, Attention deficit/hyperactivity disorder adolescence

## Abstract

**Purpose:**

To investigate the effects of socioeconomic changes from birth to 11 years of life on emotional, conduct, and attentional/hyperactivity problems in 15-year-old adolescents, from the 1993 Pelotas (Brazil) birth cohort study.

**Methods:**

The original cohort was composed of 5,249 hospital-born children whose mothers answered a questionnaire. We conducted interviews with 87.5% and 85.7% of the original cohort in 2004–2005 and 2008, respectively. We divided family income changes into nine possible categories based on two assessment points (birth and 11 years of age) and three income levels. To assess the psychopathology of the adolescents at 15 years of age, 4,423 mothers answered the Strengths and Difficulties Questionnaire.

**Results:**

Adolescents who were always poor or who became poor between birth and 11 years of age had greater conduct problems at 15 years of age. There was no consistent association between poverty and emotional and attentional/hyperactivity problems.

**Conclusions:**

The effects of income change were more specific to conduct problems than to emotional and attentional/hyperactivity problems, similar to what has been previously described in developed countries.

**Implications and Contribution**

Becoming poor from birth to adolescence was a significant determinant of conduct problems, but not of emotional or attentional/hyperactivity problems among adolescents. Preventive mental health strategies designed to tackle conduct problems in adolescents should also address the social determinants of health.

There is abundant evidence that poor children and adolescents have a high prevalence of mental disorders [1–4]. The association between family income and mental disorders starts in infancy and becomes stronger at school age and in adolescence, but varies according to the type of mental disorder [1].

Few longitudinal studies have investigated socioeconomic trajectories and their relationship to child and adolescent mental health. Existing studies suggest that persistent poverty has a greater impact than transitory poverty [5–7] and that changes in family income are associated with conduct disorders, but not with anxiety or depression [5,6,8,9].

The effects of poverty on the mental health of children and adolescents occur both directly via environmental resource constraints and indirectly through psychological influence. Poor families tend to live in more deteriorated neighborhoods [10] and children are likely to attend poor-quality schools and health services [7,11]. Indirectly, family income can affect child and adolescent psychopathology via proximal risk mechanisms such as poor parenting [2], reduced parental supervision [5], stressful events [11], maternal depression [12], family conflict, and affiliation with deviant peers [1].

There are no studies about the income trajectories and mental health of children and adolescents from low- and middle-income countries where exposure to environmental risk factors is greater compared with high-income settings [13]. Moreover, a recent systematic review of randomized, controlled trials in these countries was inconclusive concerning the effects of poverty alleviation on improvement in child and adolescent mental health, although some conditional cash transfer and asset promotion programs had mental health benefits [8].

This study aimed to assess the association between changes in family income over a period of 11 years and emotional, conduct, and attentional/hyperactivity problems in 15 year-old adolescents belonging to a Brazilian birth cohort.

## Methods

Pelotas is a town located in the extreme south of Brazil, with a population estimated at 345,179 inhabitants, 93% of whom live in the urban area. We monitored all births occurring in the five maternity clinics in the town (99% of the births occurred in hospital) in 1993. For the 5,265 children born alive, only 16 mothers could not be interviewed or refused to participate in the study. The 5,249 newborns, whose mothers lived in the urban area, were included in the cohort. The detailed methodology of this study can be found elsewhere [14]. During the perinatal study, we interviewed mothers to collect demographic, health, and socioeconomic information about the family. In 2004–2005, we found 87.5% of the original cohort (we interviewed 4,452 mothers). In 2008, we interviewed 4,349 mothers (85.7% of the original cohort) [14].

### Explanatory variable

We divided family income at birth and at 11 years of age into tertiles. We collected and summed the incomes of each family member in the previous month. Socioeconomic changes from birth to 11 years of age therefore had nine possible categories: lowest tertile at both visits (n = 890); lowest tertile at birth and intermediate tertile at 11 years of age (n = 696); lowest tertile at birth and highest tertile at 11 years of age (n = 274); intermediate tertile at birth and lowest tertile at 11 years of age (n = 368); intermediate tertile at both visits (n = 466); intermediate tertile at birth and highest tertile at 11 years of age (n = 397); highest tertile at birth and lowest tertile at 11 years of age (n = 162); highest tertile at birth and intermediate tertile at 11 years of age (n = 277); and highest tertile at both visits (n = 755).

### Outcome variable

To measure emotional (anxiety and depression symptoms), conduct (oppositional and conduct disorder symptoms) and attentional/hyperactivity (inattention and hyperactivity symptoms) problems, 4,423 mothers or those in charge of the adolescents answered the Strengths and Difficulties Questionnaire (SDQ) via face-to-face interviews when cohort members were 15 years of age. This screening questionnaire assesses mental health problems in children and adolescents in the 6 months before the interview. The SDQ was developed by Goodman [15] and validated in Brazil by Fleitlich-Bilyk and Goodman [16]. A previous study, when cohort members were 11 years of age, compared the SDQ with a diagnostic instrument (Development and Well-Being Assessment). The psychometric properties were 78.2% sensitivity, 70.4% specificity, 90.2% negative predictive value, 48.2% positive predictive value, and 74.0% area under the curve [17,18]. In the current study, we used SDQ continuous scores of emotional, conduct, and attentional/hyperactivity problems subscales.

### Confounding variables

We included the following perinatal variables in the models as possible confounders: child’s sex, smoking during pregnancy (mothers answered “yes/no” as to whether they had smoked during pregnancy), maternal marital status (single versus married/partner), schooling, and maternal age (in years).

### Potential mediating variables

We considered the following variables assessed at 11 years of age to be possible mediators (intervening factors influenced by income that also affect child mental health): stressful events and maternal mental health problems. Stressful events refer to events occurring in the previous year (death and/or serious illness of family members, and experience of racial and/or social discrimination). We assessed maternal mental health during an interview using the Self-Report Questionnaire–20. This instrument measures maternal mental health, mainly depression and anxiety symptoms (not psychotic) in the past month. It was developed by Harding et al [19] and validated in Brazil by Mari and Williams [20]. Mothers who reported eight or more symptoms were considered to be deviants (positive screening). We also collected information about skin color (white, black, mixed, native, or Asian) as self-reported when the adolescent was 11 years of age.

### Data analysis

In the bivariate analyses, we compared mean scores of conduct, emotional, and attentional/hyperactivity problems across income changes, using one-way analysis of variance. We employed linear regression in the adjusted analysis.

The adjusted analyses followed a conceptual model of determination of adolescent psychopathology. The first group of variables included the independent variable (family income change), and sociodemographic risk factors collected at birth and at 11 years of age: sex and skin color of the adolescent, smoking during mother’s pregnancy, age and schooling of the mother, and family composition. The second group of variables included the possible mediating variables: maternal mental health, mental disorder of the mother and stressful events. We conducted analyses using STATA 11.2 (Stata Corporation, College Station, TX).

The Research Ethics Committee of the Medical School of the Universidade Federal de Pelotas approved the project. Parents and youths received a detailed explanation of the procedures and signed an informed consent declaration before data collection.

## Results

The mean SDQ subscale scores were 2.38 (standard deviation [SD] = 2.26) for conduct problems, 3.94 (SD = 2.69) for emotional problems, and 3.80 (SD = 3.06) for attentional/hyperactivity problems. Table 1 presents the distribution of the sample and mean scores for conduct, emotional, and attentional/hyperactivity problems by income changes and other variables of interest. Almost half of the families did not change income group from birth to 11 years of age; almost 20% remained in the poorest tertile in this period. The prevalence of a positive score for mothers’ mental health problems was approximately 30%.

Table 2 presents the crude and adjusted regression coefficients for the association between income changes and conduct problems at 15 years of age. In the second adjusted model, those who were in the lowest income tertile at 11 years of age had higher scores for conduct problems than those who were always in the highest income tertile. Those who moved from the highest to the lowest tertile had a coefficient of .430 (95% confidence interval [CI], .052–.808), whereas those moving from the intermediate tertile to the lowest tertile had a coefficient of .555 (95% CI, .266–.845) compared with those remaining in the highest income tertile from birth to 11 years of age. Those who remained in the low-income or intermediate levels also had higher scores for conduct problems than those remaining in high-income families.

Tables 3 and 4 present results from regression analyses for emotional problems and attentional/hyperactivity scores, respectively. In the second adjusted model, only families that moved from the highest to the intermediate income tertile (β = .435 [95% CI, .074–.796]) had significantly higher emotional problem scores. For problems of attentional/hyperactivity, only those in the intermediate category (β = .369 [95% CI, .002–.735]) and those who moved from the lowest to the highest tertile (β = .450 [95% CI, .019–.881]) continued to present significantly higher scores.

**Figure 1:**
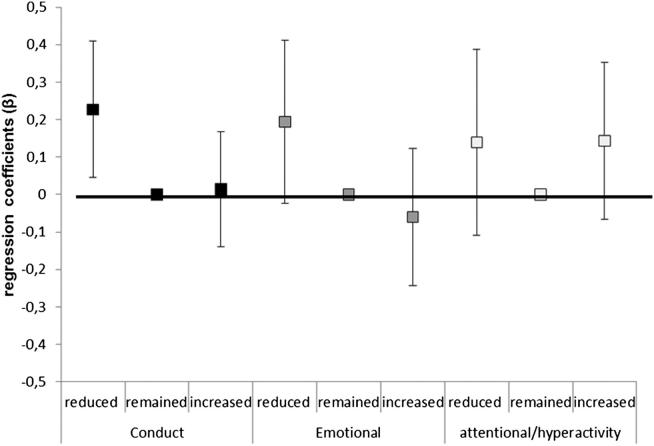
Adolescents’ conduct, emotional and attentional/hyperactivity problems at 15 years of age, according to three groups of socioeconomic changes from birth to 11 years.

Figure 1 shows levels of conduct, emotional, and attentional/hyperactivity problems according to three broader categories of family income changes: one group in whom income was reduced, a second group in whom income remained stable, and a third group for whom income increased. The only significant association was between income reduction and higher conduct problems (conduct problems: reduced *p* = .015, increased *p* = .860; emotional problems: reduced *p* = .520, increased *p* = .081; attentional problems: reduced *p* = .275, increased *p* = .179).

## Discussion

Adolescents who were poor from birth or who became poor from birth to 11 years of age had more conduct problems at 15 years of age than adolescents in continually high-income families. Emotional problems had a weaker association with poverty that was probably mediated by increased exposure to stressful events and maternal mental illness. There was no consistent association between poverty and attentional/hyperactivity problems.

Other longitudinal studies have also found trajectories of family income to be more strongly associated with conduct, oppositional, and aggressive problems than with anxiety and depression. A natural experiment assessed the mental health of adolescents before and after an intervention that improved family income. The effects of increases in family income were specific to reducing symptoms of conduct and oppositional defiant disorders, and showed no effects on anxiety and depression. Anxiety and depression symptoms were more common in poor children, but moving out of poverty was not followed by a reduction in these symptoms [5]. As a possible explanation for the specificity of effects of increased income on conduct and oppositional disorders, the authors suggested that anxiety and depression in children and adolescents may be caused by characteristics of poor families that are not directly related to poverty. Alternatively, the remarkable speed of the change in behavioral symptoms after coming out of poverty may be specific to conduct and oppositional symptoms, and effects on anxiety or depression might be more delayed [5]. Another study that observed children and adolescents for 4 years showed that poverty at study baseline affected outcomes of both depression and antisocial disorder. However, reduction in family income over 4 years only influenced the antisocial disorder outcome, increasing its symptoms [6]. Parents living in persistent poverty are under greater stress than those who experience transient poverty. Their stress may lead to harsh disciplinary practices that increase children’s risk of conduct problems. Alternatively, exposure to unsafe environmental conditions associated with poverty, such as dangerous neighborhoods, may increase children’s conduct problems [6]. A quasi-experimental study assessed mental health in children after a conditional cash transfer program. Participation in the program was associated with a 10% decrease in aggressive and oppositional symptoms among children, although there was no statistically significant program effect for symptoms of anxiety and depression. Results did not differ for boys versus girls [9]. A possible interpretation for the different results in aggressive or oppositional versus anxiety and depression symptoms is that the program may enable parents to provide more consistent structure and monitoring or supervision for their children, potentially conferring particular benefits for aggressive and oppositional/conduct problems [9].

Adolescents who were always poor in the current study (low-low) and those who became poor from birth to 11 years of age (high-low) had higher scores of conduct problems compared with those who were never poor (high-high). In contrast, the group that came out of poverty and rose to the highest income tertile at 11 years of age (low-high) did not have higher conduct problems. The results suggest the role of social determinants in the development of conduct problems in mid-adolescence. Because we evaluated income from birth to early adolescence, when participants had few direct influences on family income, the hypothesis of reverse causation (poverty springing from the fact that the individual has a mental disorder) is less probable in our study compared with studies of poverty and mental disorder among adults. There are inconsistencies in the literature concerning the relevance of timing: when children experience poverty and its impact on conduct problems. Whereas some studies found an association between poverty in infancy and antisocial behavior in adolescence [7], other studies showed that current poverty (in adolescence) was more important for influencing conduct problems [6]. Because many studies about poverty in infancy and conduct problems assessed family income in only one wave, new studies about changes in family income are required.

Our results suggest that family income changes showed a strong unadjusted association with emotional problems that remained significant after controlling for confounding sociodemographic variables (adjusted analyses 1, Table 3). However, these effects were possibly mediated by maternal mental health problems and stressful events in early adolescence, which resulted in weaker and inconsistent associations in the final models (adjusted analyses 2, Table 3). These results suggest that maternal mental health and stressful events may be potential mediators of the influence of family income changes on adolescents’ emotional problems.

Attentional/hyperactivity problems were also not consistently associated with poverty (for both adjusted analyses 1 and 2, Table 4). The only study that we found about income trajectories that included attention deficit hyperactivity disorder (ADHD) as an outcome did not report on this association owing to the low prevalence of such disorders in some family income categories [5]. Thus, it was not possible to compare the current findings on attentional/hyperactivity problems with those from other samples. The null results may have been because ADHD is a condition with strong biological and genetic determination [21]. Moreover, it is difficult to formulate specific hypothesis for some of the results—for example, the increase in attentional/hyperactivity problems scores in the group with a positive trajectory (low-high)—particularly considering that we did not assess important determinants of ADHD such as gestational, genetic, and maternal ADHD factors in this study [21,22].

### Limitations

Assessment of children’s conduct, emotional, and inattentive/hyperactive problems at baseline (perinatal, at the same time when first family income was assessed) is not logistically possible. Therefore, we cannot disentangle whether the effects of income changes on conduct problems at 15 years of age were biased by the presence of these symptoms from very early child development. In addition, antisocial behavior in parents (also not assessed) may predict income and change in income (e.g., through job instability or unemployment) as well as adolescent conduct problems.

Another limitation of our study was the use of a screening instrument for the outcome variables. However, the SDQ had an adequate psychometric performance in previous studies of this cohort [17,18]. The lack of multiple informants to evaluate mental health problems of the adolescents was also a limitation. It is known that parental reports about children’s emotional problems (anxiety and depression) are less reliable than children’s self-reports of these symptoms [23]. Because we measured the mediators at the same time as the last measurement of income and we did not conduct a formal test for mediation, these results only suggest that stressful events and maternal mental health problems are potential mediators.

The effects of negative family income changes from birth to 11 years of age were more specific to conduct problems than to emotional and attentional/hyperactivity problems in mid-adolescence. The results suggest a role of social determinants in the development of conduct problems. Our findings extend the findings from developed countries to low- and middle-income countries.

## Figures and Tables

**Table 1:** Distribution of the sample (%) and bivariate analysis of adolescent psychopathology at 15 years of age and family, maternal, and adolescent variables from birth to 11 years

Variable	%	Conduct problems (mean [SD])	*p*	Emotional problems (mean [SD])	*p*	Attentional/hyperactivity problems (mean [SD])	*p*
Family variables							
Socioeconomic status changes			<.001		<.001		<.001
Low-low	20.7	2.66 (2.41)		4.37 (2.88)		4.05 (3.01)	
Low-intermediate	16.2	2.46 (2.32)		4.07 (2.69)		3.94 (3.13)	
Low-high	6.4	2.26 (2.20)		4.01 (2.78)		4.01 (3.02)	
Intermediate-low	8.6	2.72 (2.38)		4.19 (2.60)		4.08 (3.13)	
Intermediate-intermediate	10.9	2.35 (2.35)		4.01 (2.66)		3.87 (3.21)	
Intermediate-high	9.3	1.87 (1.99)		3.42 (2.56)		3.68 (2.98)	
High-low	3.8	2.54 (2.38)		4.26 (2.82)		3.75 (2.98)	
High-intermediate	6.5	2.17 (2.08)		4.03 (2.49)		3.69 (2.98)	
High-high	17.6	1.57 (1.82)		3.27 (2.43)		3.20 (2.91)	
Stressful events			<.001		<.001		<.001
0	51.2	2.02 (2.13)		3.62 (2.58)		3.57 (3.00)	
1	35.4	2.40 (2.29)		4.07 (2.72)		3.88 (3.07)	
2 +	13.4	3.04 (2.48)		4.84 (2.80)		4.50 (3.12)	
Maternal variables							
Age (years)			<.001		.064		<.001
<20	17.4	2.84 (2.42)		4.13 (2.68)		4.40 (3.07)	
20–34	71.6	2.20 (2.22)		3.88 (2.70)		3.68 (3.03)	
≥35	11.0	1.98 (2.14)		4.01 (2.62)		3.60 (3.05)	
Schooling (years)			<.001		<.001		<.001
0–4	28.0	2.66 (2.41)		4.49 (2.82)		4.01 (2.96)	
5–8	46.2	2.38 (2.31)		3.98 (2.66)		3.93 (3.12)	
≤9	25.8	1.67 (1.85)		3.26 (2.44)		3.30 (2.98)	
Smoking during pregnancy			<.001		<.001		<.001
Yes	33.4	2.81 (2.43)		4.15 (2.68)		4.28 (3.08)	
No	66.6	2.02 (2.13)		3.83 (2.69)		3.56 (3.02)	
Mental health			<.001		<.001		<.001
Screening +	30.9	3.11 (2.42)		5.02 (2.68)		4.64 (3.03)	
Screening −	69.1	1.92 (2.09)		3.45 (2.55)		3.41 (2.99)	
Marital Status			<.001		.475		.002
Married/partner	88.1	2.23 (2.24)		3.93 (2.69)		3.74 (3.04)	
No	11.9	2.65 (2.37)		4.02 (2.69)		4.20 (3.12)	
Adolescent variables							
Sex			.002		<.001		<.001
Male	49.7	2.18 (2.20)		3.49 (2.56)		4.21 (3.14)	
Female	50.3	2.39 (2.31)		4.39 (2.73)		3.40 (2.92)	
Skin color			<.001		<.001		<.001
White	64.1	2.05 (2.13)		3.77 (2.62)		3.61 (3.02)	
Black	14.1	2.69 (2.44)		4.20 (2.81)		4.03 (3.12)	
Mixed	18.1	2.69 (2.40)		4.29 (2.78)		4.27 (3.06)	
Native/Asian	3.7	2.73 (2.36		4.23 (2.64)		3.88 (3.12)	

SD = standard deviation.

**Table 2:** Adolescents’ conduct problems at 15 years of age, according to socioeconomic changes from birth to 11 years: crude and adjusted analyses

Socioeconomic status changes	Crude analysis β (95% CI)	Adjusted analyses 1^a^ β (95% CI)	Adjusted analyses 2^b^ β (95% CI)
High-high	Reference (.000)	Reference (.000)	Reference (.000)
High-intermediate	.598 (.287–.910)	**.378** (**.067–.689)**	.288 (−.016–.593)
High-low	.967 (.585–1.350)	**.582** (**.197–.967)**	**.430** (**.052–.808)**
Intermediate-high	.297 (.024–.571)	.159 (−.114–.432)	.144 (−.123–.411)
Intermediate-intermediate	.780 (.518–1.042)	**.444** (**.175–.714)**	**.392** (**.128–.655)**
Intermediate-low	1.150 (.867–1.433)	**.713** (**.418–1.008)**	**.555** (**.266–.845)**
Low-high	.682 (.370–.993)	**.365** (**.048–.683)**	.294 (−.016–.605)
Low-intermediate	.889 (.656–1.121)	**.472** (**.221–.722)**	.**353** (**.107–.598)**
Low-low	1.085 (.864–1.306)	**.559** (**.310–.807)**	**.328** (**.082–.573)**

CI = confidence interval.

a Adjusted for sex, skin color, maternal age, schooling, smoking during pregnancy, and marital status (Model 1); adjusted R^2^ = .065.

b Adjusted for Model 1 + stressful events and mother’s mental health at age 11 years; adjusted R^2^ = .111. Bold data indicate significant (*p* < .05) findings.

**Table 3:** Adolescents’ emotional problems at 15 years of age, according to socioeconomic changes from birth to 11 years: crude and adjusted analyses

Socioeconomic status changes	Crude analysis β (95% CI)	Adjusted analyses 1^a^ β (95% CI)	Adjusted analyses 2^b^ β (95% CI)
High-high	Reference (.000)	Reference (.000)	Reference (.000)
High-intermediate	.765 (.391–1.139)	**.562** (**.189–.934)**	**.435** (**.074–.796)**
High-low	.992 (.533–1.452)	**.592** (**.131–1.053)**	.448 (−.001–.896)
Intermediate-high	.149 (−.180–.478)	−.016 (−.343–.312)	−.034 (−.351–.283)
Intermediate-intermediate	.740 (.426–1.055)	**.374** (**.051–.697)**	.284 (−.028–.598)
Intermediate-low	.926 (.585–1.266)	**.508** (**.154–.862)**	.294 (−.049–.638)
Low-high	.739 (.364–1.113)	**.468** (**.087–.848)**	.344 (−.024–.713)
Low-intermediate	.801 (.521–1.082)	**.400** (**.100–.700)**	.236 (−.055–.527)
Low-low	1.102 (.837–1.367)	**.611** (**.313–.909)**	.287 (−.004–.577)

CI = confidence interval.

a Adjusted for sex, skin color, maternal age, schooling, smoking during pregnancy, and marital status (Model 1); adjusted R^2^ = .061.

b Adjusted for Model 1 + stressful events and mother’s mental health at age 11 years; adjusted R^2^ = .126. Bold data indicate significant (*p* < .05) findings.

**Table 4:** Adolescents’ attentional/hyperactivity problems at 15 years of age, according to socioeconomic changes from birth to 11 years: crude and adjusted analyses

Socioeconomic status changes	Crude analysis β (95% CI)	Adjusted analyses 1^a^ β (95% CI)	Adjusted analyses 2^b^ β (95% CI)
High-high	Reference (.000)	Reference (.000)	Reference (.000)
High-intermediate	.494 (.067–.920)	.294 (−.133–.720)	.187 (−.235–.610)
High-low	.552 (.028–1.077)	.309 (−.219–.836)	.191 (−.334–.715)
Intermediate-high	.479 (.104–.854)	.372 (−.003–.747)	.354 (−.018–.724)
Intermediate-intermediate	.673 (.314–1.032)	**.421** (**.051–.791)**	**.369** (**.002–.735)**
Intermediate-low	.883 (.496–1.271)	**.530** (**.125–.935)**	.362 (−.040–.764)
Low-high	.812 (.385–1.239)	**.525** (**.090–.961)**	**.450** (**.019–.881)**
Low-intermediate	.746 (.426–1.065)	**.385** (**.041–.729)**	.246 (−.094–.587)
Low-low	.849 (.546–1.151)	**.391** (**.050–.733)**	.144 (−.196–.485)

CI = confidence interval.

a Adjusted for sex, skin color, maternal age, schooling, smoking during pregnancy, and marital status (Model 1); adjusted R^2^ = .041. Bold data indicate significant (*p* < .05) findings.

b Adjusted for Model 1 + stressful events and mother’s mental health at age 11 years; adjusted R^2^ = .072.
